# Combined linkage and association analysis of classical Hodgkin lymphoma

**DOI:** 10.18632/oncotarget.24872

**Published:** 2018-04-17

**Authors:** Alastair Lawrie, Shuo Han, Amit Sud, Fay Hosking, Timothee Cezard, David Turner, Caroline Clark, Graeme I. Murray, Dominic J. Culligan, Richard S. Houlston, Mark A. Vickers

**Affiliations:** ^1^ School of Medicine, Medical Sciences and Nutrition, University of Aberdeen, Aberdeen, United Kingdom; ^2^ Division of Genetics and Epidemiology, The Institute of Cancer Research, London, UK; ^3^ The Genepool, University of Edinburgh, Edinburgh, United Kingdom; ^4^ Scottish National Blood Transfusion Service, Edinburgh, United Kingdom; ^5^ Department of Medical Genetics, Aberdeen Royal Infirmary, Aberdeen, United Kingdom; ^6^ Department of Haematology, Aberdeen Royal Infirmary, Aberdeen, United Kingdom; ^7^ Current address: Clinical Trials Manager, MD Anderson Cancer Centre Investigational Cancer Therapeutics, Houston, TX, USA

**Keywords:** genetics, Hodgkin lymphoma, mutation, cancer, lymphoma

## Abstract

The heritability of classical Hodgkin lymphoma (cHL) has yet to be fully deciphered. We report a family with five members diagnosed with nodular sclerosis cHL. Genetic analysis of the family provided evidence of linkage at chromosomes 2q35-37, 3p14-22 and 21q22, with logarithm of odds score >2. We excluded the possibility of common genetic variation influencing cHL risk at regions of linkage, by analysing GWAS data from 2,201 cHL cases and 12,460 controls. Whole exome sequencing of affected family members identified the shared missense mutations p.(Arg76Gln) in *FAM107A* and p.(Thr220Ala) in *SLC26A6* at 3p21 as being predicted to impact on protein function. *FAM107A* expression was shown to be low or absent in lymphoblastoid cell lines and *SLC26A6* expression lower in lymphoblastoid cell lines derived from p.(Thr220Ala) mutation carriers. Expression of *FAM107A* and *SLC26A6* was low or absent in Hodgkin Reed-Sternberg (HRS) cell lines and in HRS cells in Hodgkin lymphoma tissue. No sequence variants were detected in *KLHDC8B*, a gene previously suggested as a cause of familial cHL linked to 3p21. Our findings provide evidence for candidate gene susceptibility to familial cHL.

## INTRODUCTION

Classical Hodgkin lymphoma (cHL) is the commonest lymphoid malignancy of young adults [1]. While cHL results from the neoplastic transformation of germinal centre B-cells, the characteristic Hodgkin and Reed-Sternberg (HRS) cells are typically seen at very low levels (1–10%), with most of the tumour consisting of a pleomorphic infiltrate but predominantly T-lymphocytes [2–4].

Epstein-Barr virus (EBV) infection is causally related to a number of cases [3, 5], but there is little evidence to support the involvement of other environmental or lifestyle risk factors. A role for inherited susceptibility to cHL is provided by familial risk [6, 7], including a high concordance in monozygotic twins [8]. Although a strong human leukocyte antigen (HLA) association for cHL risk is established [9–14], this only accounts for <30% of the familial risk [15]. Direct evidence for the role of non-HLA genetics in cHL has come from recent genome-wide association studies (GWAS), which have identified common variants at 13 loci associated with the risk of sporadic cHL [9, 14, 16, 17]. In addition to common genetic variation influencing cHL, reports of familial aggregation raise the possibility of the existence of Mendelian susceptibility to the disease, caused by the inheritance of high-impact mutations [18]. A number of these multiple-case families have been the subject of linkage searches and various putative linkage signals reported; notably at 3p21.31 implicating germline variation in the kelch protein gene *KLHDC8B* [19].

Nodular sclerosis Hodgkin lymphoma (NSHL), the most common histological subtype of cHL in developed countries, is more frequent in young adults and women [4]. With the aim of furthering our understanding of cHL susceptibility, we describe a family in which five members have been diagnosed with the cHL subtype, NSHL. Seeking to identify a major risk locus responsible for cHL in the family, we conducted a genome-wide linkage scan and whole exome sequencing (WES) of affected family members. To complement the analysis, we also searched for evidence of association at regions of linkage by analysing a large GWAS dataset.

## RESULTS

### Linkage and mutation analysis

No region of the genome showed evidence for linkage with cHL in the family at genome-wide significance (*i.e*. logarithm of odds (LOD) score >3.0). Three regions of the genome, however showed evidence of linkage with a LOD score greater than 2.0: 2q35-37 (220-237Mb), 3p14-22 (42-66Mb), 21q22 (42-47Mb) (Supplementary Figure 1). Linkage at 6p21 (HLA) was lacking and no shared HLA class I allele was shown between affected family members (Table 1). Previous studies have demonstrated a strong association between HLA class II alleles with EBV-negative NSHL. Only the HLA-DPB1*04:01 allele (Table 1), which has population frequency (>40%), was shared between affected family members. 712 coding variants were shared by affected family members (III-a, IV-b, IV-f and IV-g). Of these 712, only 37 mapped to the three regions of linkage. Restricting our analysis to those variants with a population frequency <1% and those predicted to impact on the protein function by both SIFT [20] and POLYPHEN-2 [21] algorithms, we identified p.(Arg76Gln) in *FAM107A* and p.(Thr220Ala) in *SLC26A6* mapping to 3p21 as two plausible candidate variants causal for cHL in the family. We confirmed the fidelity of WES for both variants by Sanger sequencing. Finally, we did not identify any rare sequence variants in the previously identified candidate cHL susceptibility gene in this region, *KLHDC8B,* in any of the affected family members.

**Table 1:** Clinico-pathological details of affected family members

ID	Stage	Treatment	Age at diagnosis	HLA type	Subtype, EBV status
IV-f	2A	6 × ABVD	22	A*02:01,A*02:01, B*07:02,B*13:02, C*06:02,C*07:02, DRB1*07:01,DRB1*15:01, DRB4*01, DRB5*01, DQB1*02:02,DQB1*06:02, DPB1*04:01:01,DPB1*04:02	NSHL, LMP1 negative.
IV-b	2A	6 × ABVD, radiotherapy	18	A*01:01,A*02:01, B*08:01,B*44:02, C*05:01,C*07:01, DRB1*01:01,DRB1*03:01, DRB3*01, DQB1*05:01,DQB1*02:01, DPB1*04:01:01	NSHL, unknown
III-g	2A	3 × ABVD, radiotherapy	31	A*03:01, B*07:02,B*49:01, C*07:01,C*07:02, DRB1*04:01,DRB1*15:01, DRB4*01, DRB5*01, DQB1*06:02,DQB1*03:01, DPB1*04:01	NSHL, unknown
IV-g	4B	2 × ABVD, 6 × BEACOPPe, IFRT	25	A*03:01,A*25:01, B*07:02,B*18:01, C*07:02,C*12:03, DRB1*15:01, DRB5*01:01, DQB1*06:02, DPB1*04:01,DPB1*145:01	NSHL, EBER negative.
III-a	NA	NA	33	NA	NA

ABVD, adriamycin, bleomycin, vinblastine, dacarbazine; BEACOPP, bleomycin, etoposide, cyclophosphamide, adriamycin, vincristine, procarbazine, prednisolone; IFRT, involved-field radiotherapy, LMP1, latent membrane protein 1; EBV, Epstein–Barr virus; EBER, - Epstein–Barr virus-encoded small RNAs; NSHL, nodular sclerosis Hodgkin lymphoma; NA, not available.

### Analyses of candidate genes

**Figure 1:**
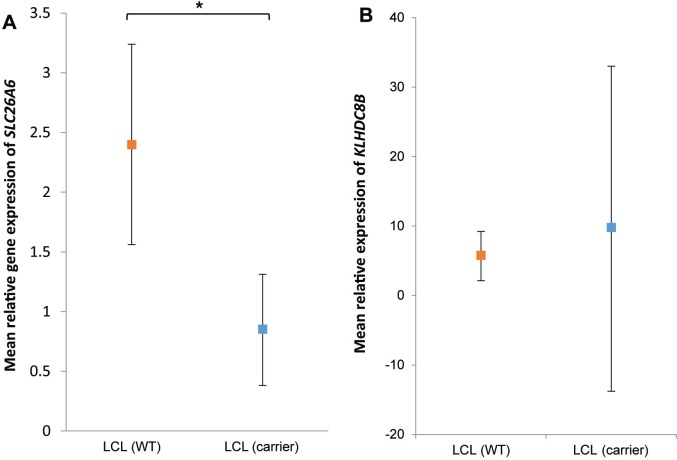
(**A**) *SLC26A6* and (**B**) *KLHDC8B* expression in lymphoblastoid cells from patients and controls. Mean relative expression in lymphoblastoid cell lines derived from mutations carriers III-g, IV-f and IV-g (blue) and ten non-carriers (red). Cell lines assayed in triplicate and normalized against GAPDH expression. Values are mean of replicates and carrier status. Error bars denote 95% confidence intervals.^*^P < 0.05.

**Figure 2:**
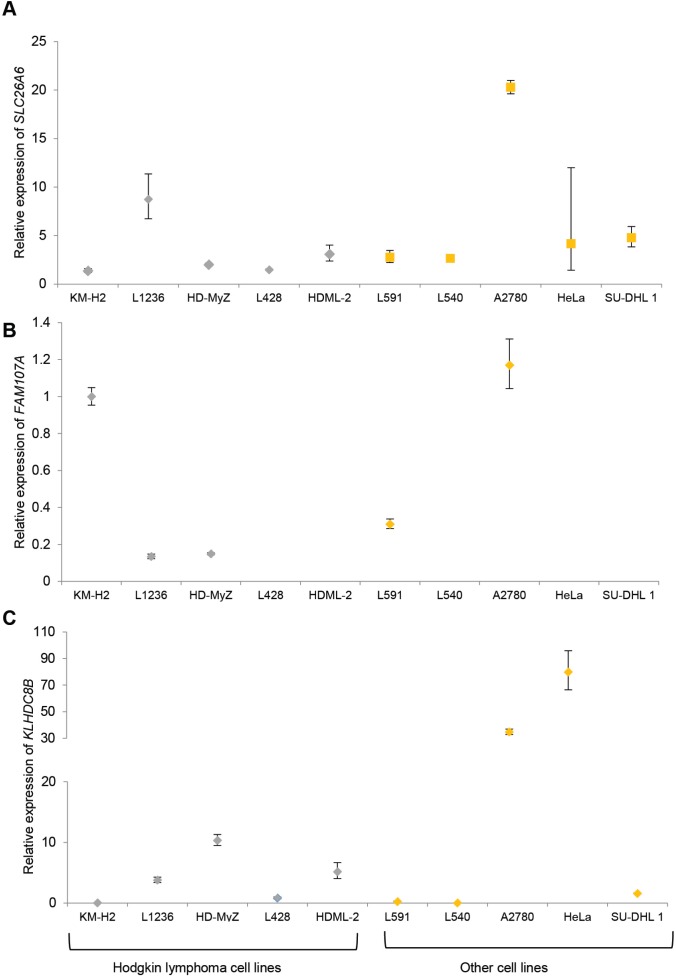
(**A**) *SLC26A6* (**B**) *FAM107A* (**C**) *KLHDC8B* expression in established cell lines. Hodgkin lymphoma cell lines: KM-H2, L1236, HD-MyZ, L428, HDML-2. Each cell line was assayed in triplicate and normalized against GAPDH expression. Expression of *FAM107A* in L428, HDML-2, L540, HeLa and SU-DHL1 was below the limit of detection. Data are shown as mean (± standard error of the mean).

**Figure 3:**
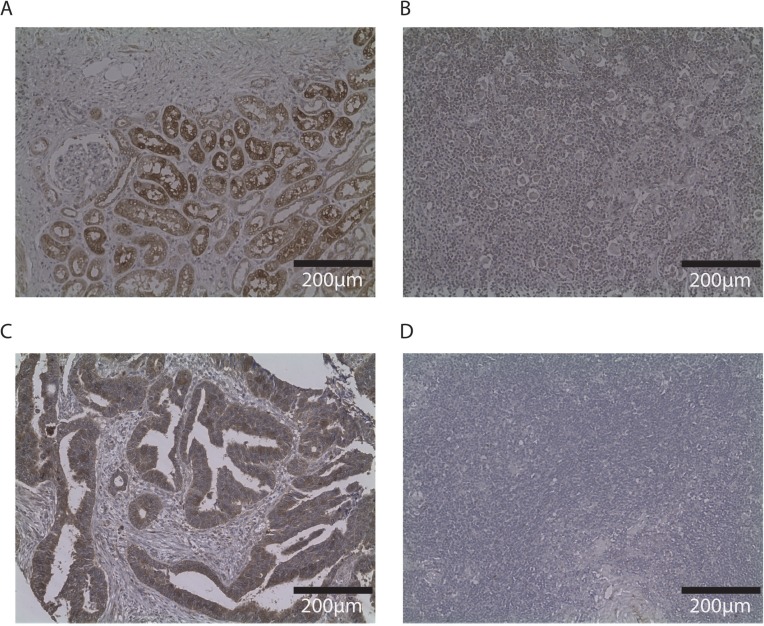
Immunohistochemistry of SLC26A6 and FAM107A in other tissues and Hodgkin lymphoma node biopsy Photos (20×) of representative examples illustrating SLC26A6 staining of renal biopsy (**A**) and Hodgkin node (**B**); FAM107A staining of colorectal cancer tissue (**C**) and Hodgkin node (**D**). Immunostaining indicated by DAB, counterstained with haematoxylin.

*SLC26A6* expression was lower in lymphoblastoid cell lines from family members carrying the T220A mutation compared to unrelated healthy individuals (Figure 1). *FAM107A* expression was undetectable in lymphoblastoid cell lines. Expression of *FAM107A* and *SLC26A6* was low or absent in HRS cell lines (Figure 2) and in HRS cells in Hodgkin lymphoma (Figure 3). Finally, expression levels of *KLHDC8B* were similar between lymphoblastoid cell lines from mutation carriers and non-carriers as well as between HRS cell lines and other cell lines (Figures 1 and 2).

### Association analysis of 2q35-37, 3p14-22, 21q22

To explore the possibility that variants with frequency greater than 1% might contribute to a relationship between 2q35-37, 3p14-22, 21q22 and cHL risk, we analysed directly typed and imputed genotypes in our large case-control series. Across the three regions we examined the relationship for 107,472 SNPs and cHL risk (Supplementary Figure 2). The strongest associations at 2q35-37, 3p14-22, 21q22 were provided by rs6714255 (*P* = 5.79 × 10^–6^), rs115918946 (*P* = 1.70 × 10^–4^) and rs2839384 (*P* = 1.31 × 10^–3^), which, after adjustment for multiple testing, were not statistically significant (*i.e*. *P* < 4.65 × 10^–7^, 0.05/107,472). Furthermore, these common genetic variants were not in linkage disequilibrium with the candidate genetic mutations in *FAM107A* and *SLC26A6*.

## DISCUSSION

It is notable that we identified the class II HLA allele (DPB1*0401) as being shared between affected individuals. Although a HLA class II association is well established for NSHL, there is limited evidence to implicate this specific HLA allele. Excluding the possibility of multiplicative interaction with a non-HLA linked allele, the pattern of inheritance of cHL in the family we describe is parsimonious with autosomal inheritance with incomplete penetrance. Predicated on this assumption, we sought to identify a disease locus for cHL in the family by performing a linkage scan. Although not statistically significant *per se,* we found suggestive linkage at 3p21, a region previously implicated by Salipante *et al.* [19], which in a combined analysis would provide a LOD score of >3.0.

By performing WES of affected individuals we identified two rare protein-disrupting mutations in *SLC26A6* and *FAM107A* on chromosome 3p21, as possible candidates for cHL risk. In contrast, we did not identify any mutations in *KLHDC8B,* which has been previously suggested as a cause of familial cHL [19], and which lies within the linkage peak on 3p21 only ∼500kb from *SLC26A6*.

*SLC26A6* is expressed in many tissues, consistent with its role as an anion transporter [22, 23]. *SLC26A6* has so far not been directly implicated in cancer. *FAM107A,* originally identified in a commonly deleted region on 3p21 in renal cell carcinoma, appears to function as a tumour suppressor [24–26]. Loss of heterozygosity at 3p21 is common in human malignancies [24, 27–30], although the region has not been shown to be recurrently lost or gained in primary HRS cells [31, 32]. However, both cHL and non-Hodgkin lymphoma have been reported to display chromosomal rearrangements involving this region by fluorescent *in situ* hybridization [33]. Furthermore, the International Cancer Genome Consortium report that mutations in *SLC26A6* and *FAM107A* occur in 3.7% (9/241) and 12.9% (31/241) respectively of germinal centre B-cell derived lymphomas [34]. Although we were able to demonstrate lower *SLC26A6* mRNA expression in LCLs of affected individuals, we have been unable to provide a mechanistic basis by which identified variants in either *SLC26A6* or *FAM107A* contributes to lymphomagenesis.

Although WES is a well-recognised strategy to identify disease-causing mutations [35, 36], it does not identify pathogenic non-coding abnormalities, which are increasingly recognised to be important [37, 38]. Hence, we cannot exclude the possibility that susceptibility to cHL in the family might be mediated through non-coding changes within the linked region. Reference to GWAS data has however provided evidence that common genetic variation does not underscore the linkage signal at 3p21. In addition, this study would be further enhanced by analysis of data relating to unaffected individuals in this family.

In summary, our analysis of this family support the existence of a susceptibility locus on chromosome 3p21 for cHL, with exome sequencing suggesting *SLC26A6* and *FAM107A* as possible candidate genes.

## MATERIALS AND METHODS

### Hodgkin lymphoma family

Collection of blood samples and clinical information from subjects was undertaken with informed consent and relevant ethical review board approval (North of Scotland regional ethics committee (12/NS/0105), in accordance with the tenets of the Declaration of Helsinki.

**Figure 4:**
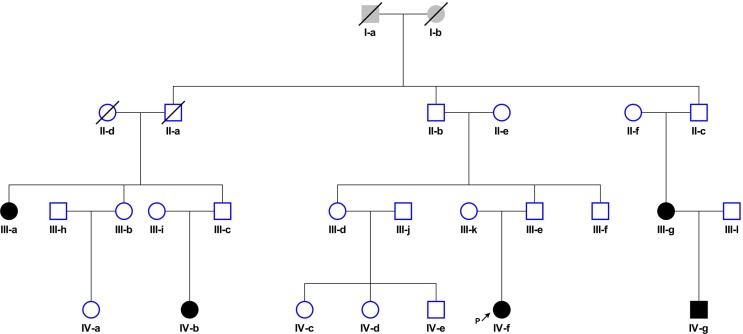
Family segregating cHL: Circles, female and squares, male Filled-in symbols represent individuals with nodular sclerosis Hodgkin lymphoma. Slash indicates individual is deceased. Arrow indicates proband.

A family segregating cHL was identified through a female patient (IV-f) diagnosed with NSHL at age 22 under the clinical care of the Department of Haematology at Aberdeen Royal Infirmary, UK (Figure 4). A detailed family history taken from this index case revealed that three members of her extended family had also been diagnosed with NSHL (III-a; III-g, IV-b), all before age 35 (Table 1). During the time the family has been under investigation, IV-g was diagnosed with stage 4B cHL at age 25. For four of the five affected family members (III-g, IV-f, IV-b, IV-g) the diagnosis of NSHL was confirmed by histological and immunohistochemical studies through reference to patient notes and diagnostic pathology. Tumors from IV-f and IV-g were EBV-negative (Table 1) by immunohistochemistry (LMP1) or *in situ* hybridization (EBER). In all family members, complete remission was attained with first-line standard treatment (*i.e*. chemotherapy ± radiotherapy). None had a significant medical history or past history of cancers and IV-f was found to have a normal constitutional chromosome karyotype.

### Genetic analysis of affected family members

Constitutional DNA was extracted from EDTA-venous blood or saliva samples using either QiaAMP DNA mini kits (Qiagen, UK) or prep-IT purification (DNAGenotek, Canada) kits and quantified by Quant-IT Picogreen (Invitrogen, UK). Family members were genotyped using either Affymetrix 6.0 chips (III-g, IV-b, IV-f, IV-g) or Affymetrix 500K (III-a) mapping arrays. CEL intensity files were processed using Affymetrix Power Tools (APT v1.16.1) and PLINK used to harmonise genotypes. Mendelian errors were removed and SNPs with high heterozygosity spaced ∼0.3Mb apart selected using linkdatagen were used for linkage analysis [39]. Non-parametric linkage analysis was performed using MERLIN [40].

Typing of classical HLA alleles was performed using SeCore sequencing (Life Technologies, UK) in conjunction with Applied Biosystems 3130XL technology and analysed using uTYPE 6.0 software (Life Technologies) with IMGT as reference (version 3.14.0).

For WES of germline DNA from III-a, IV-b, IV-f and IV-g was fragmented using a Covaris E Series instrument (Covaris, United States). Indexed paired-end libraries were prepared using the SureSelect Human All Exon 50Mb (Agilent, United States) and 2 × 100 bp sequencing performed using Illumina HiSeq2000 technology (Illumina, United States). Paired end fastq files were extracted using CASAVA software (v.1.8.1, Illumina) and aligned to build 37 (hg19) of the human reference genome using Stampy and BWA software [41, 42]. Alignments were processed using the Genome Analysis Tool Kit (GATK) pipeline [43]. We imposed GATK internal calling thresholds and required a genotyping quality (GQ) of ≥ 30. Only non-silent variants were considered for analysis (*i.e*. missense, nonsense, frameshift, in-frame insertion/deletions, splice donor/acceptors, and initiator codon variants). Exome Variant Server (NHLBI GO Exome Sequencing Project (ESP) [44], 1000 genomes project [45] and dbSNP [46] were used as population frequency references. Functional consequences of missense changes were predicted using SIFT [20] and POLYPHEN [21] algorithms. Sanger confirmation of sequence changes was carried out by using Big-Dye Ver 3.1 chemistry implemented on an ABI3730xl (Applied Biosystems, Foster City, USA).

### Detailed studies of *SLC26A6* and *FAM107A*

Immunohistochemistry of SLC26A6 and FAM107A were performed on formalin-fixed, paraffin-embedded material lymph node biopsies from IV-f and sporadic cHL (*n* = 5) patients using primary antibodies to SLC26A6 (Sigma) or FAM107A (Biorbyt) and DAB chromogen (Dako).

*SLC26A6, KLHDC8B* and *FAM107A* RNA expression was evaluated in lymphoblastoid cell lines (LCLs) derived from III-g, IV-f and IV-g and healthy controls (*n* = 10), HL cells (L428, L1236, HD-MyZ, KM-H2, HDML-2) and other cell lines (L540, L591, A2780, HeLa, SU-DHL1). RNA was extracted using the RNeasy mini kit (Qiagen, UK) and qRT-PCR performed using a Roche LightCycler 480 thermal cycler. Assays were performed in triplicate with *GAPDH* as internal control.

### Association at 2q35-37, 3p14-22, 21q22

To evaluate the association between common genetic variation at chromosomes 2q35-37, 3p14-22, 21q22 and cHL we made use of data previously generated on two non-overlapping case-control series of Northern European ancestry, which had been the subject of a previous GWAS [14]. The UK GWAS was based on 589 cases ascertained through Royal Marsden Hospitals National Health Service Trust Family History study during 2004–2008. Individuals from the 1958 Birth Cohort and National Blood Service served as controls [47]. The UK-NSHLG GWAS was based on 1,612 cases ascertained through the National Study of Hodgkin lymphoma Genetics [14]. Individuals from the BCAC and PRACTICAL consortia served as controls [48]. Collection of blood samples and clinical information from subjects was undertaken with informed consent and relevant ethical review board approval in accordance with the tenets of the Declaration of Helsinki. Full details of the genotyping and quality control are detailed in previously published work [14]. Briefly, we have previously confirmed an absence of systematic genetic differences between cases and controls and shown no significant evidence of population stratification in these sample sets. GWAS data were imputed to >10 million SNP with IMPUTE2 v2.392 software [49], using a merged reference panel consisting of data from 1000 Genomes Project (phase 1 integrated release 3, March 2012) [45] and UK10K (ALSPAC, EGAS00001000090/EGAD00001000195 and TwinsUK EGAS00001000108/EGAS00001000194 studies) [50]. Tests of association between SNP genotype and cHL were performed under an additive genetic model in SNPTESTv2.5 [51]. Meta-analyses were performed under a fixed-effects model using META v1.6101 [52].

## SUPPLEMENTARY MATERIALS FIGURES


