# Supplementary material for: Combined linkage and association analysis of classical Hodgkin lymphoma
Source: Oncotarget. 2018 Apr 17;9(29):20377–85. doi: 10.18632/oncotarget.24872 (PMC5945548; doi:10.18632/oncotarget.24872)
Supplement: Supplementary file 1 [file oncotarget-09-20377-s001.pdf]

Combined linkage and association analysis of classical Hodgkin lymphoma

SUPPLEMENTARY MATERIALS

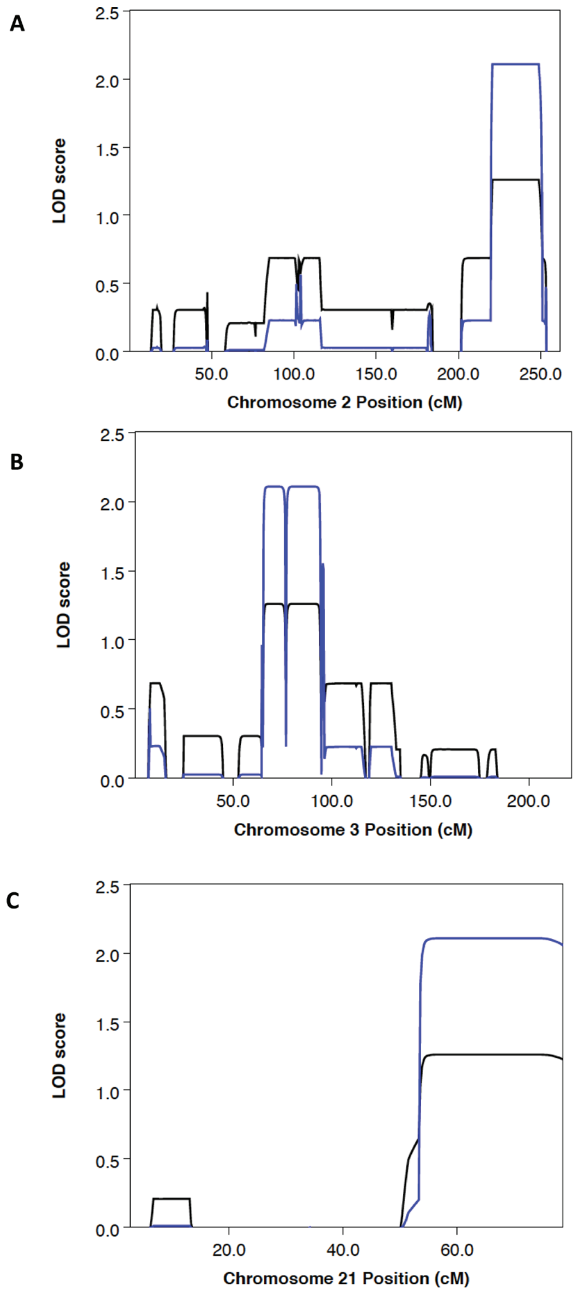

Supplementary Figure 1: LOD scores in cHL family at (**A**) chromosome 2, (**B**) chromosome 3 and (**C**) chromosome 21. LOD scores calculated using the Kong and Cox linear (black) exponential (blue) model.

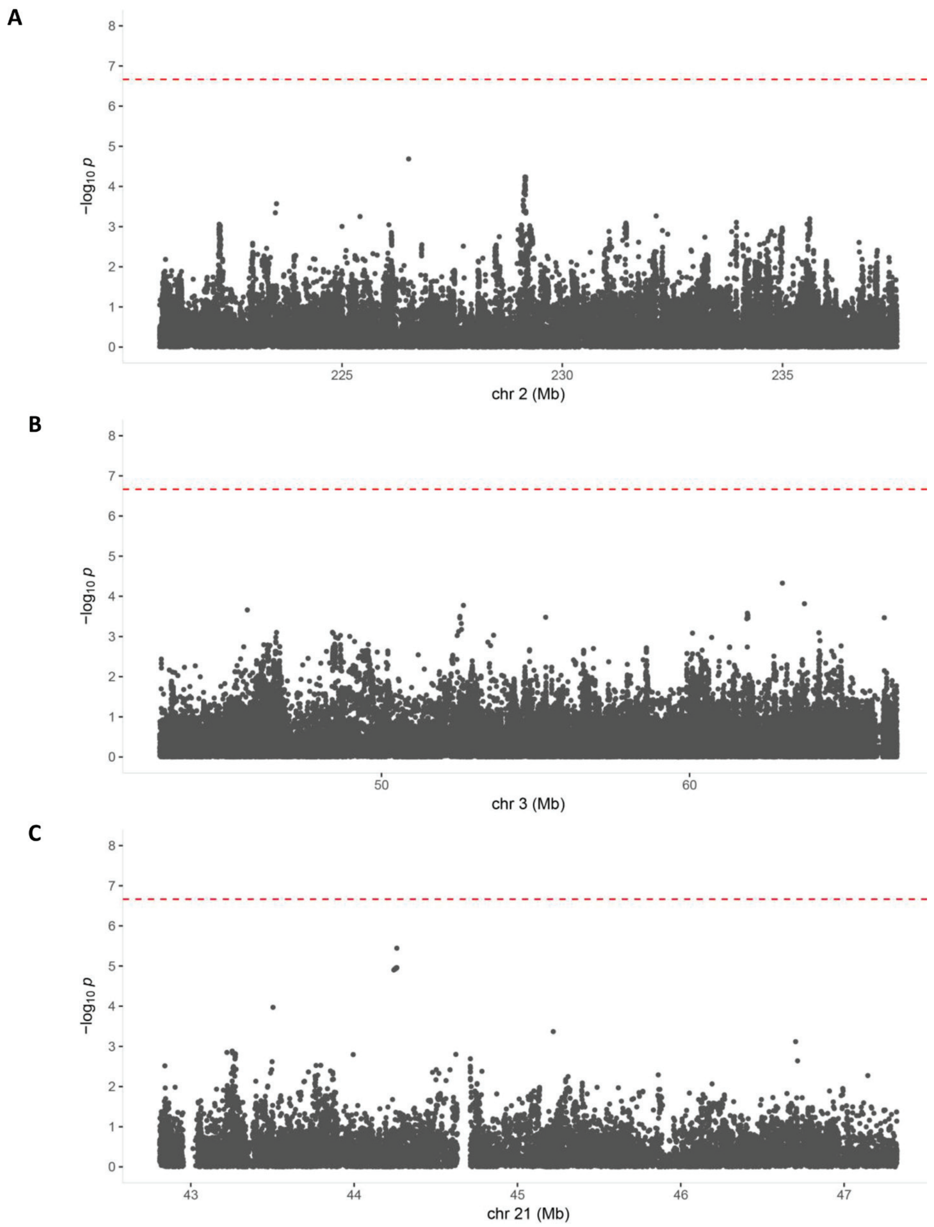

Supplementary Figure 2: Regional plots of association at regions of linkage (**A**) chromosome 2:220,861,593–237,597,846 bp; (**B**) chromosome 3:42,800,778–66,729,080 bp (**C**) chromosome 21:42,809, 206–47,323,742 bp. $-\log_{10}$ *P*-values (*y* axes) of the SNPs are shown according to their NCBI build 37 chromosomal positions (*x* axes). The red horizontal line represents the significance threshold of $P = 4.65 \times 10^{-7}$.
